# UVB-Aged Microplastics and Cellular Damage: An in Vitro Study

**DOI:** 10.1007/s00244-024-01073-x

**Published:** 2024-06-19

**Authors:** Sebastiano La Maestra, Mirko Benvenuti, Stefano Alberti, Linda Ferrea, Francesco D’Agostini

**Affiliations:** 1 Department of Health Sciences, University of Genoa, Via A. Pastore, 1, 16132 Genoa, Italy; 2 Department of Chemistry and Industrial Chemistry, University of Genoa, Via Dodecaneso 31, 16146 Genoa, Italy

## Abstract

Plastics are synthetic organic compounds whose widespread use generates enormous waste. Different processes, such as mechanical abrasion, microbiological activity, and UVB irradiation, can fragment the plastic material and generate microplastics (MPs). MPs are ubiquitous, and various organisms, including humans, can ingest or inhale them, with potential adverse health effects. The differences between UV-aged and virgin particles were studied to evaluate the genotoxic damage and oxidative stress induced by polystyrene MPs with 1 and 5 µm sizes on the monocyte-like cell line (THP-1). Fourier transform infrared spectroscopy and Ζ-potential measurements were used to characterise MP particles after UVB exposure. Cells exposed to MPs show a widespread change in the cellular environment with the generation of reactive oxidative species (ROS), as indicated by the increased malondialdehyde level. The occurrence of genotoxic damage is correlated to the smaller size and ageing state of the MPs. The biochemical and genomic alterations observed in this in vitro study suggest that MPs, ubiquitous pollutants, following natural degradation and oxidation processes can cause various adverse effects on the health of the exposed population, making it necessary to carry out further studies to better define the real risk.

Plastics are organic polymeric compounds synthesised from natural resources such as gas, oil, and its derivatives. These polymers have many applications in different sectors, including packaging, construction, automotive, and electronics. Due to their advantageous characteristics and low production costs, manufacturing increases yearly (Hu et al. 2023). Extensive use generates enormous quantities of plastic waste. It has been calculated that from 1950 to 2015, about 6.3 billion tons of plastic waste were generated, and 26 billion tons will be produced by 2050 (Geyer et al. 2017; Guglielmi 2017).

Commonly used plastics are polyethylene terephthalate (PET), polypropylene (PP), polyethylene (PE), polyvinyl chloride (PVC), polystyrene (PS), and polyurethane (PU). All these polymers are characterised by environmental persistence due to their molecular stability, which are due to relatively stable covalent bonds involving C–C and C-H groups. These groups may undergo degradation reactions triggered by heat and light (UVB) (Wilkinson et al. 2017), causing instability and breaking into small fragments. Moreover, environmental MPs are degraded by different metabolic microorganisms (Miri et al. 2022). Depending on their size, plastic particles can be classified as MPs with dimensions between 0.001 and 5 mm and nanoplastics (NPs) smaller than 0.1 µm (Kubowicz and Booth 2017).

MP pollutants can be generated by mechanical abrasion (Song et al. 2017) weathering on environmentally dispersed macroplastics or by releasing small fragments from clothing, car tyres, paint coatings, pre-production dust, pellet spills, or also found in various preparations, such as cosmetic products (Vethaak and Legler 2021).

According to their source, MPs can be classified into primary or secondary. Primary MPs originate as small particles released directly into the environment. They are derived from textiles, medical products, and personal care products. Secondary MPs result from the degradation process of large amounts of plastic waste (Eriksen et al. 2013; Alomar et al. 2017).

Due to their small size, MPs can be considered ubiquitous and carried through the marine and terrestrial environment and into the atmosphere (Lim 2021). Moreover, their small size makes them easily ingested by aquatic organisms perpetuated throughout the trophic chain (Lim 2021; Desforges et al. 2015; Nelms et al. 2018).

Although MPs have also been found in different environmental matrices, such as drinking water, WHO (2019) stated that there are not enough studies to define the toxicity of these particles on human health. On the other hand, humans may ingest or inhale MPs, potentially affecting health (Vethaak and Legler 2021). Once ingested, MPs may cross the gastrointestinal tract epithelium gaps to disseminate into the circulatory system to be transported to the lymph nodes, liver, and spleen. Several subjects showed the presence of MPs < 2 µm in blood and different organs, such as the human placenta's foetal, maternal, and chorioamnionitis membrane sides (Ragusa et al. 2021), although it is unclear whether MPs with larger sizes follow the same pathways (Revel et al. 2018). Inhaled MPs with a size < 2.5 μm can cross the respiratory barrier (Liao et al. 2011) by alveolar macrophages to disseminate at the systemic level (Vethaak and Leslie 2016).

MPs have been observed to lead to intestinal barrier dysfunction, microbiota imbalance, alteration in triglyceride synthesis and lipogenesis, and reduction in intestinal mucus secretion (Rahman et al. 2021). Ingested MPs can be eliminated in the stool or urine (Massardo et al. 2024). A recent study suggests that the renal system can eliminate MPs above 10 nm in the urine after glomerulus filtration. MPs with larger dimensions could cross the renal tubules by exocytosis from the efferent artery and endocytosis from the proximal convoluted tubule (Pironti et al. 2022).

On the other hand, ingested MPs can cross the gastrointestinal system, damage the epithelia, or be evacuated through the faeces (Yan et al. 2022). Moreover, MPs can carry a series of toxic and other polluted compounds that could increase the risk of adverse effects (Rist et al. 2018). In vitro studies showed that MPs induce oxidative stress, damaging mitochondrial membranes, generating unbalanced membrane potential (Wang et al. 2021). ROS underlying the genotoxicity processes are involved in the multi-step process of carcinogenesis (De Sá Junior et al. 2017) and chronic disease. Human studies reported that MPs and NPs may induce DNA damage, such as micronuclei (MN) formation, chromosomal aberration, DNA strand breaks, and genotoxicity (Ballesteros et al. 2020).

The present study aims to assess genotoxic damage and oxidative stress induced by polystyrene MPs 1 and 5 µm in size on THP-1 cells to reproduce the systemic effect when the particles are taken by ingestion or inhalation and to identify the different effects between UV-aged and non-aged particles. Different assays were performed to assess cytotoxicity, oxidative stress, genotoxicity, and aneuploidy.

## Materials and Methods

### Chemicals and Reagents

All reagents as salts, grown medium, foetal bovine serum, 3-(4,5-dimethylthiazol-2-yl)-2,5-diphenyltetrazolium bromide, MDA standard, cytochalasin B, antibiotics, Schiff’s reagent, and ethidium bromide were purchased by Sigma-Aldrich (St. Louis, Missouri, USA).

### Microplastics Oxidation

PS virgin MPs of 1 µm and 5 µm were acquired by Cospheric (Santa Barbara, California 93,160 USA), and their oxidation was performed by UVB lamp exposure at 318 nm (57 V, 20 W G13, Philips) started in the presence of 40% hydrogen peroxide (Batel et al. 2016). In particular, microplastic suspensions (10 mg/ml) were placed in a glass disc containing an equal volume of H_2_O_2_, UVB-irradiated for 96 h and periodically agitated. At the end of treatment, the MPs suspension was precipitated by centrifuging (12,000 rpm for 10 min), washed in Milli-Q water, and dried by SpeedVac (Jouan RC 10.10 Heated Evaporative Centrifuge Concentrator, Milano, Italy). Precipitated microplastics were weighted and resuspended in phosphate buffer saline (PBS) at known concentrations. Before each use, the microplastic samples were resuspended using ultrasonic devices (Bandelin SONOREX™ SUPER, Ultrasonic baths, 35 kHz, Berlin, Germany).

### ζ-potential

Ζ-potential (surface charge) measurements on microplastic samples were performed using Dynamic Light Scattering (DLS) analyses, with a Zetasizer Instrument, Nano ZS90 Series (Malvern Panalytical, Malvern, UK), working with He–Ne laser (emission λ = 633 nm). Samples were subjected to 40 kHz sonication before each analysis. Measurements were performed at a fixed temperature (T = 25 °C) using a Peltier thermostatic system, with an equilibrating time set to 120 s. MPs were dispersed in deionised water to reach a final concentration of 0.1 mg mL^−1^. Measurements were performed in triplicate, and each measurement consisted of 20 runs.

## Fourier Transform Infrared Spectroscopy

Fourier transform infrared spectroscopy (FTIR) measurements were performed on Perkin Elmer Spectrum 65 FTIR, which was equipped with an attenuated total reflectance (ATR) accessory (Waltham, MA, USA). The spectra were collected in the range of 4,000–500 cm^−1^; each spectrum results from 10 accumulations of 15 s each.

The spectra obtained from FTIR analysis were used to determine the carbonyl index (CI) as reported by different studies (Zhang et al. 2021; Hu et al. 2023) and calculated as the ratio between the absorbance of the carbonyl peak and the absorbance of the methylene peak, considered as reference. In particular, for PS samples, the carbonyl peak is usually calculated at 1,730 cm^−1^ and the methylene at 1,452 cm^−1^. Furthermore, the same analysis allowed us to calculate the oxidation index (I_ox_) (Vicente et al. 2009) as:

$${I}_{ox}=\frac{\int {Abs(t)}_{C=O}}{\int {Abs(t)}_{ref}}-\frac{\int {Abs(0)}_{C=O}}{\int {Abs(0)}_{ref}}$$

where:

Abs(t)_C=O_ absorbance of C = O (1,615–1,840 cm^−1^) at reaction time t.

Abs(t)_ref_ absorbance of reference peaks (1,471–1,522 cm^−1^) at reaction time t.

Abs(0)_C=O_ absorbance of C = O (1,615–1,840 cm^−1^) for unmodified polystyrene.

Abs(0)_ref_ absorbance of reference peaks (1,471–1,522 cm^−1^) for unmodified polystyrene.

This parameter can be used as a reference for the ageing of the material under study, and it quantifies this process as a function of the oxidised groups.

### Cell Culture

The human monocytic leukaemia cell line (THP-1) was obtained from the European Collection of Cell Cultures (ECACC). Monocytes derived from peripheral blood patients with acute monocytic leukaemia were used to mimic the toxicological responses of systemic monocytes/macrophages. Briefly, the THP-1 cell was maintained in a complete culture medium (RPMI, 10% FBS, 10% Glutamine, and 1% Penicillin/Streptomycin) and split every two days or used for different assays.

### Cell Viability

MTT (3-[4,5-dimethylthiazol-2-yl]-2,5 diphenyl tetrazolium bromide) assay measures the insoluble formazan derived from tetrazolium salt and generated by the activity of mitochondria of viable cells. The assay was performed in triplicate, and the cells were cultured for 24 h in 96-well microplates (1.5 × 10^4^). Eight replicates were used for each tested concentration (25, 50, 100, 250, and 500 μg mL^−1^) of oxidised or virgin microplastics with sizes of 5 and 1 μm at two different exposure times (24 and 48 h).

After exposure, 0.5 mg mL^−1^ of MTT was added to each well, and the microplates were reincubated at 37 °C for 3 h. Dimethyl sulphoxide (DMSO) was used to solubilise the purple-coloured formazan crystals, proportional to the number of metabolically active cells. Measurement was performed at 570 nm using a microplate spectro‐photometer reader (Tecan Italia, Milan, Italy). The values obtained were compared to the negative control, and 100% vitality was assigned.

### Comet Assay

The alkaline comet assay was performed using a protocol suggested by Singh et al. (1988) with some modifications (La Maestra et al. 2020*)*. Briefly, the THP-1 cells were seeded in 96-well plates at the density of 1.5 × 10^4^ cells/well and exposed to microplastic, both oxidised and non-oxidised at two different sizes (1 and 5 µm), and two concentrations (25 and 50 μg mL^−1^) per 24 h. After incubation, the viability of cells was checked by trypan blue exclusion test. A less than 80% viability was considered an exclusion factor for the sample. After that, about 20,000 cells were embedded in 75 μL of 0.5% low melting-point agarose, coated onto slides, covered with a coverslip, and allowed to solidify at 4 °C, followed by a second layer of low melting-point agarose.

The slides were immersed in cold lysis solutions (2.5 M NaCl, 100 mM ethylenediaminetetraacetic acid, 10 mM Tris, pH 10, 1% Triton X-100, and 10% dimethyl sulphoxide) overnight, rinsed in alkaline solution (0.3 M NaOH, 1 mM ethylenediaminetetraacetic acid, pH 13), and placed horizontally in an electrophoresis chamber (Bio-Rad, Italy, Milan) in which it was performed, in fresh alkaline solution, electrophoresis (30 min at 25 V (0.66 V/cm), adjusted to 300 mA). After electrophoresis, the slides were gently removed and washed in a neutralisation buffer (0.4 M Tris–HCl, pH 7.5) for 5 min and stained with bromide iodide (2 µg mL^−1^). One hundred random nuclei were acquired with a fluorescence microscope and a digital camera at a magnification of 200 × . The analysis was conducted using CASP (Comet assay software project, http://www.casp.sourceforge.net), and the results were expressed in terms of the percentage of DNA in the tail (TDNA %). Statistical analyses were performed using ANOVA followed by Bonferroni’s t test for the multi-group comparisons test; P < 0.05 was considered significant for all tests.

### Cytokinesis-Block Micronucleus Assay

Cytokinesis-block micronucleus assay (CBMN) was performed in duplicate to highlight breakage or loss of chromosomes following exposure to microplastic, as described by Fenech (2000). Briefly, 10 × 10^4^ THP-1 cells were seeded onto 15 ml tubes and exposed to equal microplastics and concentrations used for the comet assay. After 24 h exposure, cells were added with 4 µg/mL cytochalasin B. THP-1 cells were maintained in culture for an additional 28 h and then washed and resuspended in 0.075 M KCl hypotonic solution for 2 min, centrifugated at 1,200 rpm for 10 min and prefixed in 3:5 methanol/acetic acid, and washed twice with a 6:1 methanol/acetic acid fixative solution.

Microscopic slides were obtained by smearing each sample. Subsequently, the slides underwent acid hydrolysis for 1 h ( HCl 5 N), rinsed in distilled water and deoxyribonucleic acid specifically stained with Schiff’s reagent (Sigma Chemical Co., St. Louis, MO) for 30 min, washed in distilled water, and left for 5 min in running tap water in order to intensify the pink colour. Finally, the samples were washed, blotted dry, and mounted. One thousand cells from each sample were examined under an optical microscope at 1000 × magnification to score the presence of micronucleated cells and binucleate cells. Fisher’s exact test was performed to determine a statistically significant difference between different treatments and the negative control.

### Malondialdehyde (MDA)

Briefly, 15 × 10^4^ THP-1 cells were seeded onto 15 ml tubes and exposed to concentrations MPs reported above. After 24 h exposure, the cells were collected by centrifuging 10 min at 2,200 rpm, and each cell aliquot was tested for lipid peroxidation by TBARS reaction as described by Ohkawa et al. (1979). Therefore, 200 µl of 8.1% sodium dodecyl sulphate, 1,500 µl of 20% acetic acid solution (pH 3.5), and 1,500 µl of 0.8% aqueous solution thiobarbituric acid were added to the cells. Distilled water was added to reach a volume of 4 ml, and the samples were heated at 95 °C for 60 min. After, 4 ml of n-butanol and pyridine (15:1 v/v) were added, shaken vigorously, and centrifugated at 4,000 rpm for 10 min. The organic layer was taken, and a fluorometric measurement (HITACHI, Tokyo, Japan) was made (Ex 533 nm; Em 553 nm). Moreover, an external standard, MDA, was used, and the results were expressed as nanomoles MDA equivalent per cell number.

### Statistical Analyses

The analyses were performed by JMP software (version 17. SAS Institute Inc., Cary, NC, 1989–2023). The results regarding multiple individual experiments were expressed as means ± SD, and data were analysed by one-way analysis of variance (ANOVA) with post hoc testing using the Bonferroni test. A *P* value of < 0.05 was considered as statistically significant. Fisher’s exact test was performed with a statistically significant difference between each treatment and the control with respect to the frequency of micronucleated cells.

## Results

### Fourier Transform Infrared Spectroscopy (FTIR)

**Fig. 1:**
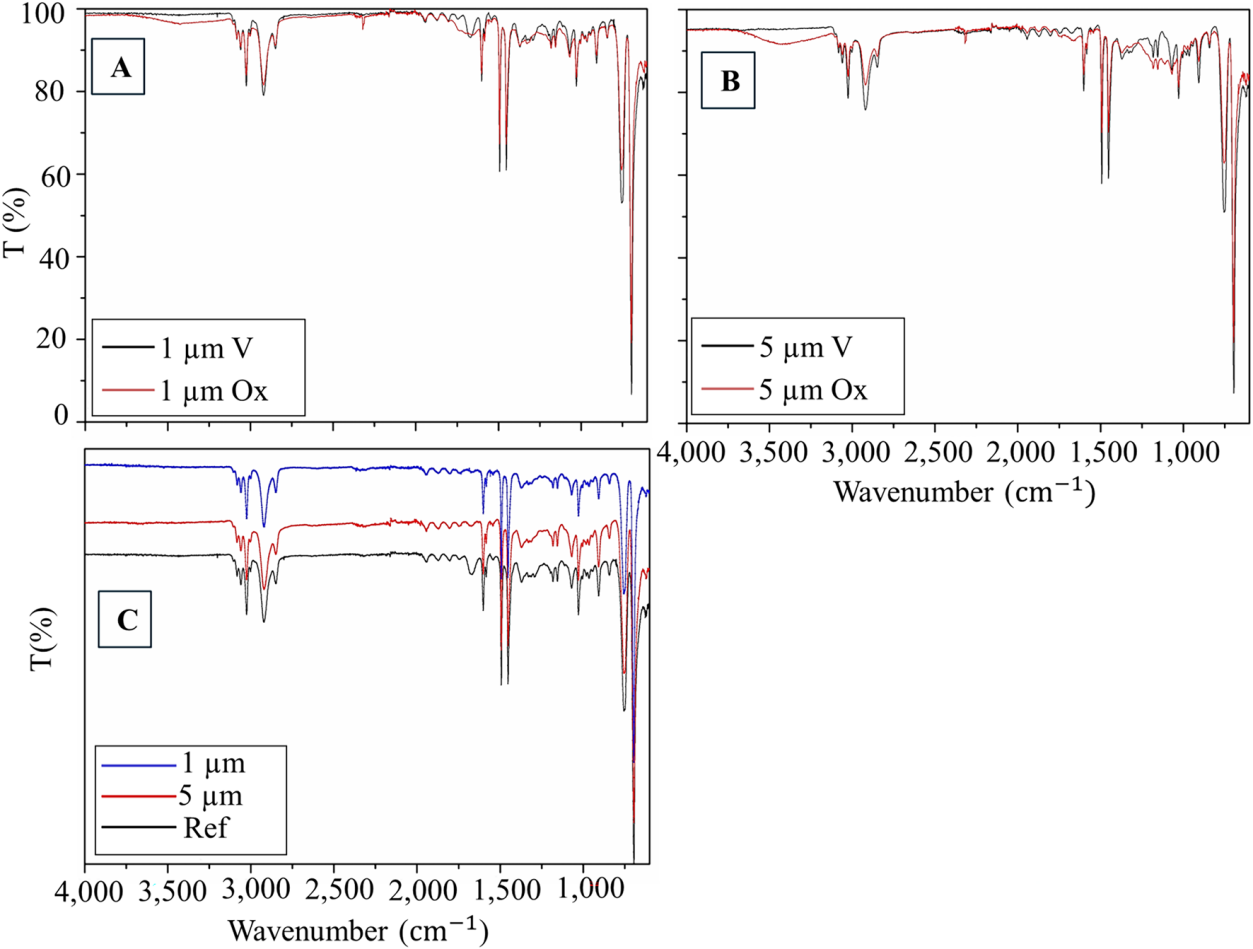
FTIR spectra in the MPs range 4,000–600 cm^−1^. **a** *black* line 1 μm V and *red* 1 μm Ox; **b** *black* line 5 μm V and *red* 5 μm Ox; **c** comparison of MPs reference spectra with different MPs samples

Figure 1a, b reports FTIR spectra obtained for virgin and oxidated MPs of both dimensions, 1 μm and 5 μm MPs, respectively. In contrast, Fig. 1c reports the comparison of virgin samples with PS standard reference spectrum.

The analyses of the MPs highlighted a broad band centred around 3500 cm^−1^, only in the photo-aged PS, typical of the stretching vibration of the O–H groups. This band most likely reflects the increase in the number of oxygenated features. Furthermore, at the wavelength of 1650–1750 cm^−1^ and 1100–1350 cm^−1^ it is possible to report peaks attributable to C = O vibrations, as reported by (Hu et al. 2023).

All spectra detect signals around 3020 cm^−1^, due to the stretching vibration of the C-H bond of the aromatic groups, along with additional signals at 2910 cm^−1^, due to the stretching of the C-H bonds of the methylene groups. The presence of aromatic groups is further highlighted with the set of low but typical signals between 1750 and 2100 cm^−1^, which are also slightly affected by the ageing process, likely due to the oxidation of the aromatic group.

**Fig. 2:**
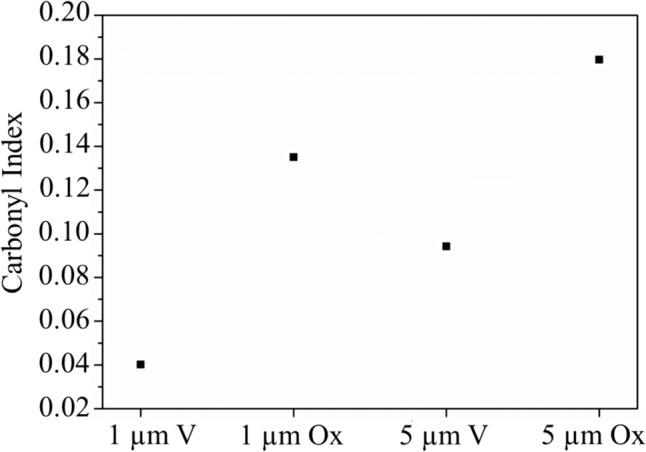
Carbonyl index of MPs samples

Figure 2 shows the evolution of carbonyl index (CI), highlighting an increase in the index value as the ageing process progressed for both PS sizes. This result provides further evidence of the oxidation process, particularly of the mechanisms that consider the presence of oxygen-containing functional groups due to the UV ageing action.

As further evidence of the oxidation process, the I_ox_, as suggested by Vicente et al. (2009), was roughly calculated for aged samples and compared with a standard PS sample (Table 1). Even though an absolute range of I_ox_ values is unavailable (I_ox_ values are reported between 0,0 and 2,0), we can see the difference between the aged samples and the standard PS.

**Table 1:** I_ox_ value in UVB ageing MPs

Sample	I_ox_
PS 1 μm	0,95
PS 5 μm	0,90
Standard PS	0,00

### ζ-potential

**Fig. 3:**
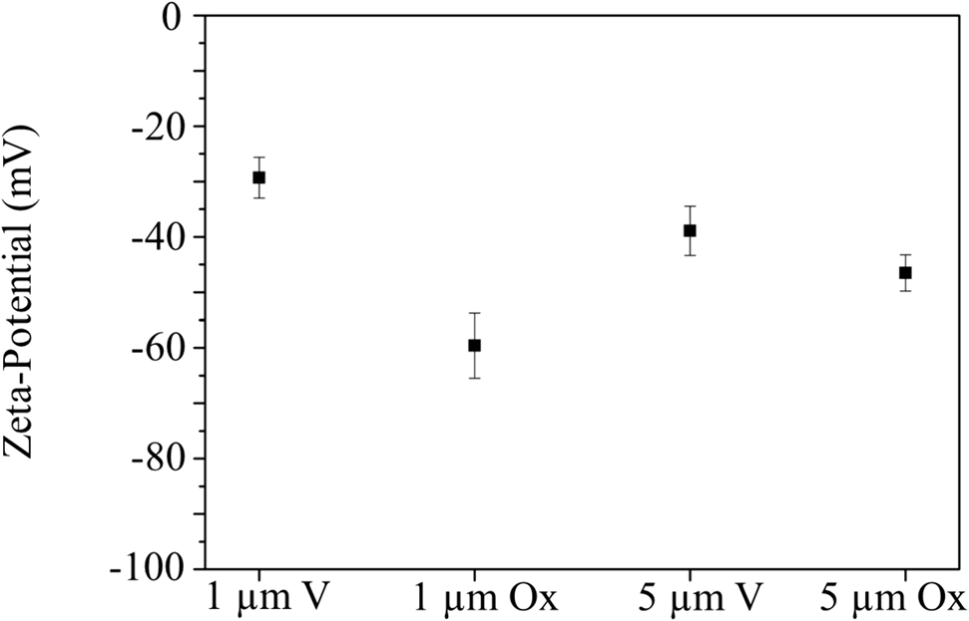
Z-potential measurement in investigated samples

Finally, Z-potential values found for investigated samples are reported in Fig. 3. Even though values do not agree with the ones reported by the manufacturer (specifications about the experimental set-up were not reported, so the measurement conditions are unknown), Z-pot values are negative for all samples.

Aged samples present a much negative value of Z-pot, which can be safely attributed to more oxygen-containing functional groups that make the surface of the MPs more negative. This outcome confirms the presence of a different surface condition characterising aged MPs concerning the bare samples.

### Cell Viability

MTT assays performed on THP-1 cells at increasing doses of microplastics (25, 50, 100, 250, and 500 µg/mL), both oxidised and virgin, with contact times of 24H and 48 h, did not show any effect on viability, even at higher doses and at longer exposure times. For this reason, the results were not reported in the text.

### DNA Damage

**Fig. 4:**
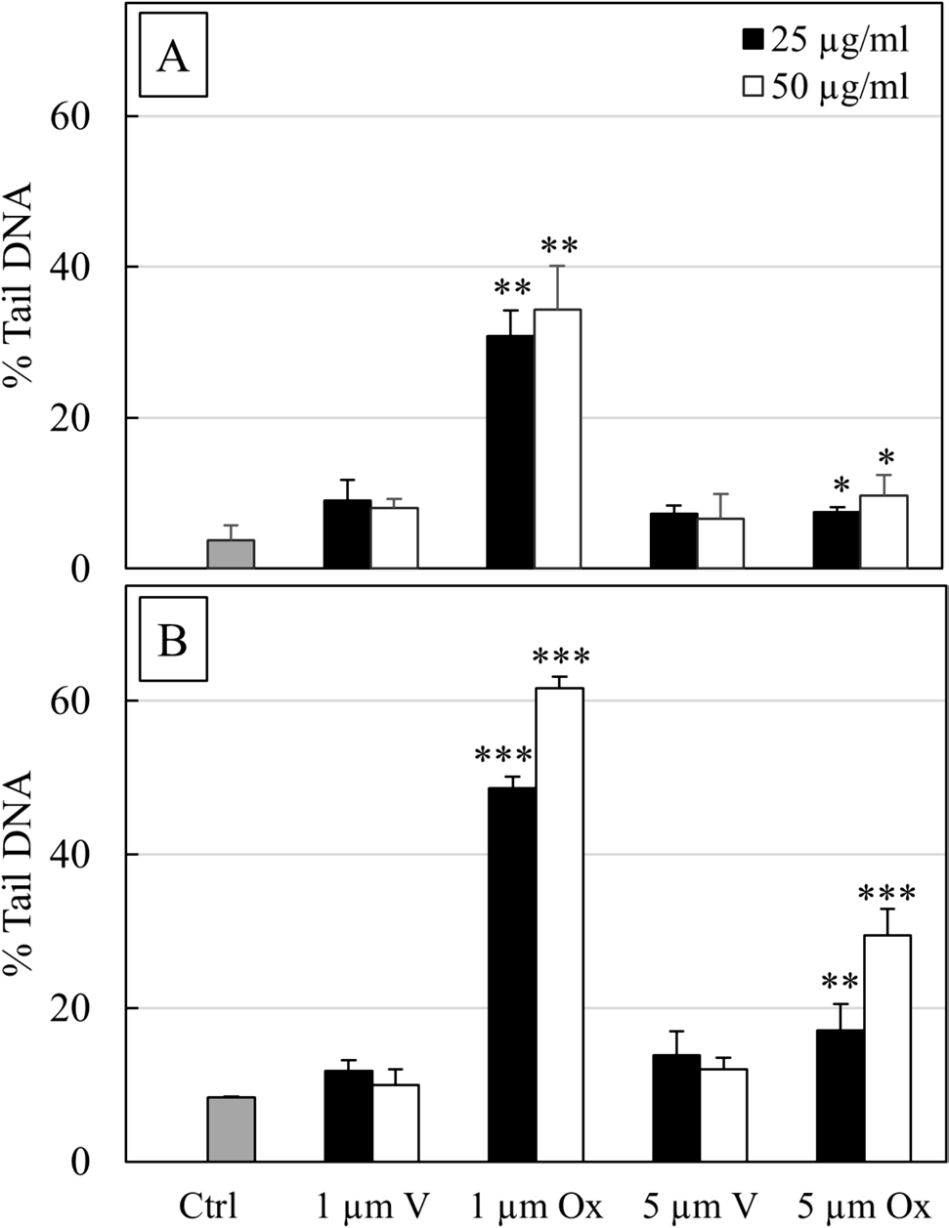
DNA damage (% TDNA) obtained by comet assay in THP-1 cells when exposed to 1 µm and 5 µm MPs, oxidate (ox) or virgin (v) at two different concentrations (25 and 50 µg/ml) and two times of contact (24 (**a**) and 48 h (**b**)). The columns report the means + SD of triplicate analyses. Statistical analysis: *P < 0.05, **P < 0.01 and ***P < 0.001 vs. controls

**Fig. 5:**
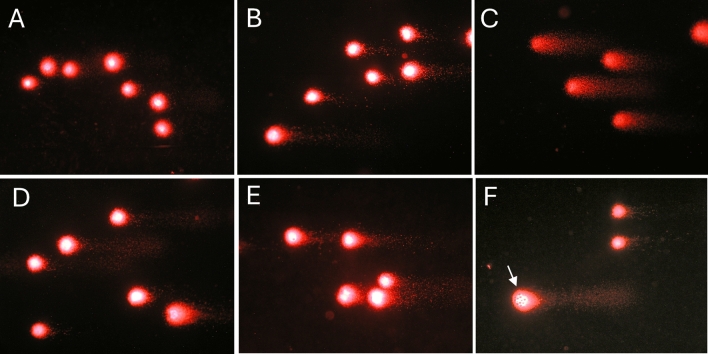
Representative photomicrograph of DNA damage obtained by comet assay in THP-1 cells when exposed to 1 µm and 5 µm MPs, oxidate (ox) or virgin (v) for 48 h. **a** Ctrl; **b** 1 µm V; **c** 1 µm OX; **d** 5 µm V; **e** 5 µm OX; **f** Example of 5 µm OX MPs incorporated within the nuclear matrix (arrow). 200X magnification

Comet assay was performed to evaluate genotoxic damage induced by exposition of THP-1 cells at two different sizes of MPs (1 and 5 µm) and concentrations (25 and 50 µg/ml) when oxidated by UVB exposition or in their native state. Genotoxic damage in THP-1 (Fig. 4) was expressed as a percentage of DNA in the tail (%TailDNA) after 24 h or 48 h of exposure, and the results were compared with untreated cells (Ctrl). Representative pictures of DNA damage obtained by comet assay are reported in Fig. 5.

The assay showed a significant increase in %TailDNA when the cells were exposed to MPs. In particular, after 24 h, cells exposed to 1 µm MPs_ox_ showed 5.9-fold DNA damage in the samples treated with 25 µg/ml and 7.2 in the samples treated with 50 µg/ml (P < 0.001). A moderate increase in damage (P < 0.05) was reported in all cells exposed to 1 µm MPs_v_, with fold variations of 2.4 at 25 µg/ml and 2.2 at 50 µg/ml. When the cells were exposed to 5 µm MPs, an increase in DNA damage was observed but with a lower magnitude. In general, the DNA damage reached a fold change around 2 (P < 0.05) in all samples tested. Forty-eight hours of exposition showed a similar DNA damage trend, albeit the magnitude was higher in all tested samples, as shown in Fig. 4.

### Micronuclei

**Fig. 6:**
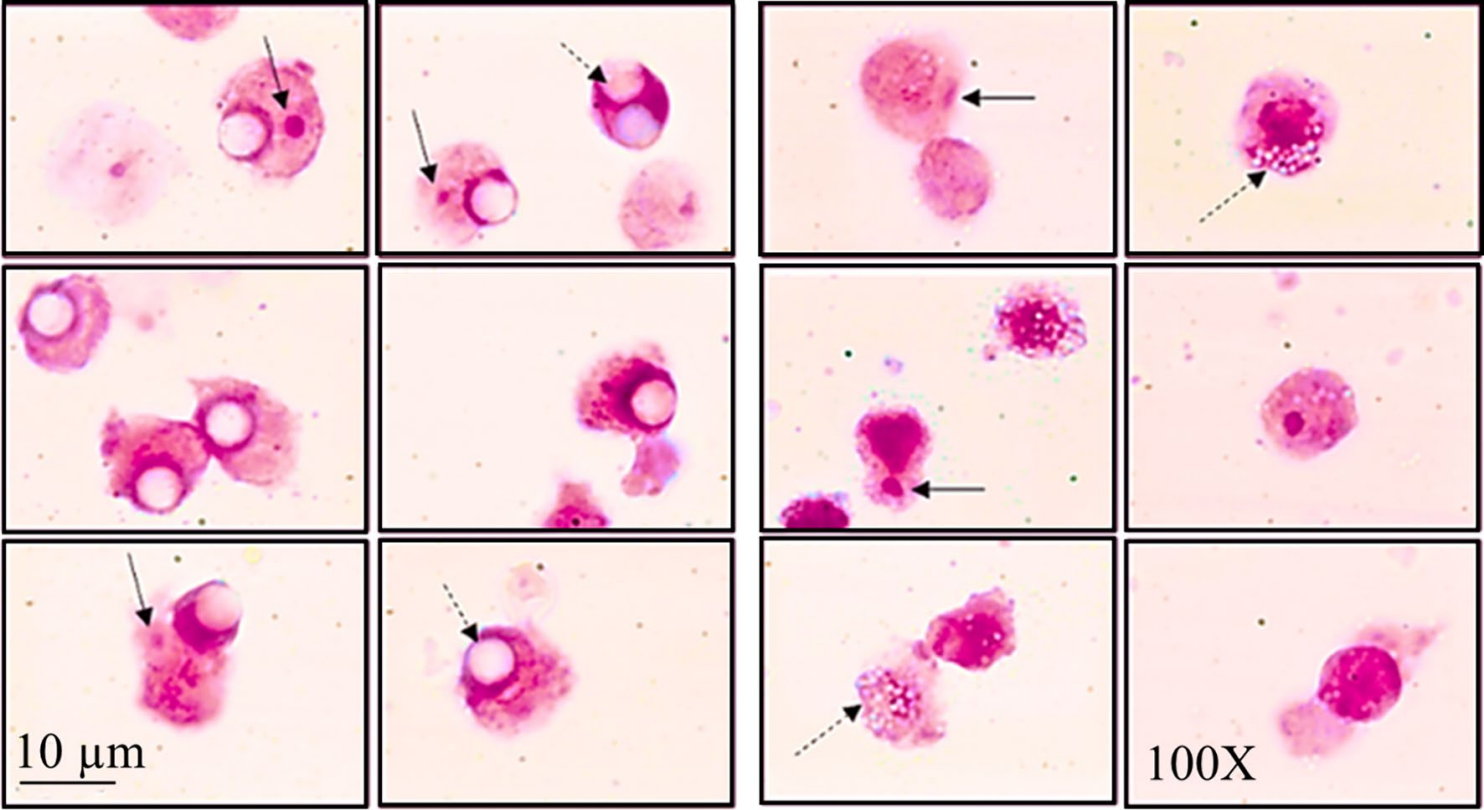
Photomicrographs obtained under an optical microscope representative of THP-1 cells exposed to 5 µm MPs (*right panel*) and 1 µm MPs (*left panel*), respectively. In the image, the intracellular MPs are indicated by dashed arrows, while solid arrows indicate the presence of micronuclei. 1,000X magnification

The photomicrograph in Fig. 6 illustrates examples of internalisation of MPs in THP-1 cells and, in some of these cells, examples of micronucleus formation.

**Fig. 7:**
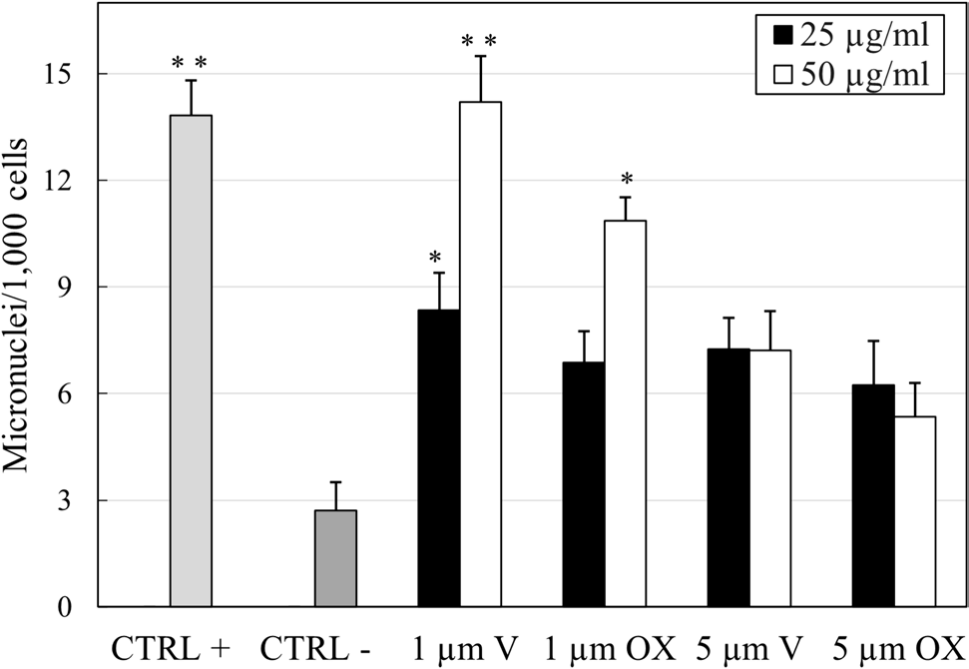
Frequency of micronuclei (MN), cytokinesis-block proliferation index (CBPI) in THP-1 cell lines after 24 h of contact, either untreated (CTRL-) or exposed to methanesulphonate (CTRL +) or MPs of varying size (1 or 5 µm) when oxidated or virgin. Statistical analysis performed by Fisher’s exact test: *P < 0.05, **P < 0.01 as compared with the negative control

Figure 7 shows MN cell frequency after treatment with different MPs. Positive control cells exposed to methyl methanesulphonate showed a considerable increase in MN (P < 0.01).

Treatment with MPs in some treatments elicited a significant increase in the frequency of MN, although there were no significant differences relative to the negative control in the treatments with oxidated or virgin MPs in the 5 µm size range (Fig. 7). A higher statistically significant change was observed in THP-1 cells exposed to 1 µm MPs_V_ with a dose-dependent response. In particular, cells treated with 1 µm MPs_V_ at 25 µg/ml reported a fold change of 3.1 (P < 0.05) when at 50 µg/ml of fivefold (P < 0.01). Micronucleus formation increased in cells in contact with 1 µm MPs_ox_ at higher concentration as fourfold change (P < 0.05).

### MDA

**Fig. 8:**
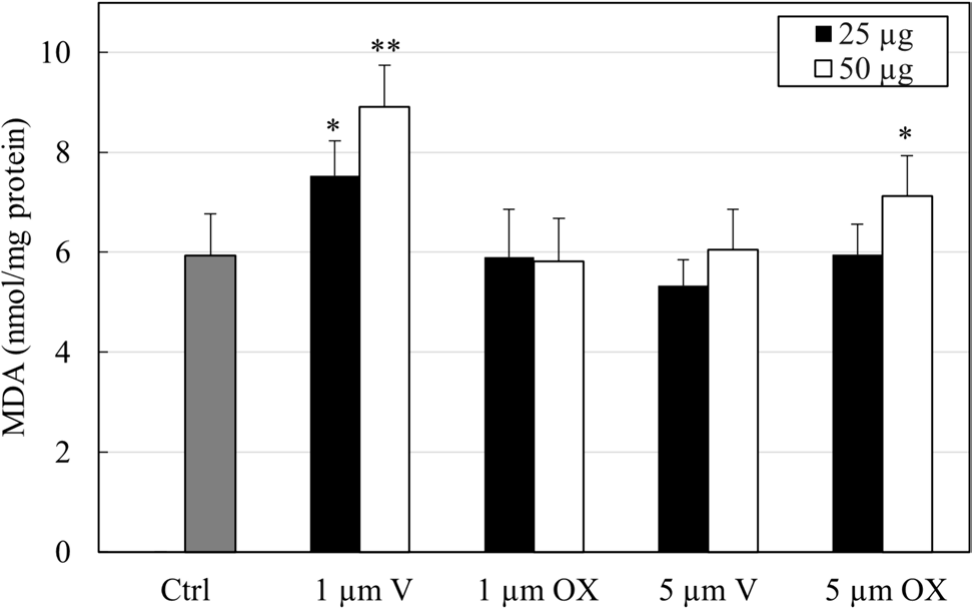
MDA levels, expressed as nmol/mg protein, in THP-1 cells after contact for 24 h with two concentrations of MPs (25 and 50 µg/ml) of different size (5 or 1 µm) and status (virgin or oxidate). The columns report the means + SD of triplicate analyses. Statistical analysis: *P < 0.05 and **P < 0.01 vs. controls

Figure 8 shows the marker of lipid peroxidation expressed as MDA levels obtained by TBARS reaction. A significant increase was reported in THP-1 when exposed to 1 μm MPs_V_ at concentrations of 25 µg (*P* < 0.05) and 50 µg (*P* < 0.01). Differently, a significant increase was only at the concentration of 50 µg for MPsOx 5 µm (*P* < 0.05).

## Discussion

The results of the present study show that UVB triggers oxidative processes that alter MPs surface structure. Ageing changes their morphology, colour, size, and surface charge. (Hu et al. 2023).

The laboratory-based model for UVB ageing simulated the solar irradiation of plastic particles dispersed in the environmental water. UVB ageing process can increase the release of toxic components such as phthalates and bisphenol-A (Weis and Alava 2023), or increased absorption of environmental organic pollutants enhances the toxicity of MPs dispersed in different environmental matrices (Bhagat et al. 2022).

PS is reported to have a negative surface charge, which increases after ageing (Pelley et al*.,* 2008), as obtained by our measure. This behaviour may be explained by the oxidation of the carbon atoms induced by hydroxyl groups to carbonyl, carboxylic, and carbon dioxide (Zhang et al. 2021).

The particle surface charge is one of the critical determinants of biological injury that influences cellular uptake efficiency through other factors such as aggregation/agglomeration, protein corona formation, and composition (Jeon et al. 2018).

Our in vitro study evaluated the effects of both oxidated and virgin MPs at two different sizes in THP-1 cell line to reproduce the systemic effect when the particles are taken by ingestion or inhalation. The results indicate the fact that MPs are able to generate widespread damage in the cellular environment. The action of MPs depends mainly on size, surface features, and dose. In fact, although our viability test has not highlighted a significant mortality increase at the different concentrations tested, an impairment of oxidative stress and genotoxic damage was identified after MPs cell exposure.

In particular, ingested or inhaled MPs can cross the epithelial barrier, diffuse into the circulatory or lymphatic systems, and, by macrophages, deposit in different organs such as the intestine, liver, and kidney (Barceló et al. 2023). MPs can cross membrane cells, triggering negative consequences in different cellular structures. The observed redox imbalance, expressed as MDA generation, reflects the ability of small-size MPs_v_ to generate ROS. In this case, the generation of ROS in the cellular environment can be due to mitochondrial membrane damage. In fact, it was observed that MDA increases as the MPs sizes decrease (Jeong et al. 2016). Furthermore, ROS generation can be attributed to direct lysosomal damage induced by attempts of these organelles to digest the foreign body or to the excessive production of intracellular ROS that can denature lysosome membranes.

PS is a stable, inert material that has a slight negative charge. Following oxidative processes, the surface charge acquires a more negative potential. The enrichment of charges determines more electrochemical interactions, facilitating bonds with serum proteins and triggering protein corona formation. Corona structures critically impact biological systems, offering new identities to MPs, such as cellular internalisation and interaction targets (Cao et al. 2022). Indeed, genotoxic tests indicate that the deleterious effects of MPs are mainly due to their smaller size and ageing state. Specifically, the comet test results show that oxidised particles are more capable of causing DNA damage. This could be related to the more significant negative surface charges on MPs ox, which promote greater interaction with serum proteins, increasing their bio-availability. Indeed, it is believed that both the primary physicochemical properties of MPs and those acquired in biological systems play a predominant role in cellular pathogenesis.

On the other hand, the aneugenic and clastogenic effects observed in THP-1 cells after MPs exposure highlight that the particles of 1 µm increase the genomic instability. The most plausible hypothesis is that the segregation error can most likely be attributable to the disturbance that the microparticles cause in the organisation of the mitotic spindle. These effects were reported by Çobanoğlu et al., (2021), who observed an increase in MN frequency in human peripheral blood lymphocytes exposed to PE in vitro. The same author highlights that MPs can cause genetic instabilities after chronic exposure. However, it is worth highlighting that the studies conducted by Çobanoğlu link the MN increase to PE exposure, which, having a different chemical composition from PS, could lead to different mechanical interactions. This highlights the need for further studies to better understand the mechanisms through which MPs cause MN formation (Shamy et al. 2002; Somorovská et al. 1999; Ballesteros et al. 2020; Laffon et al. 2002).

MPs represent a ubiquitous pollutant of significant importance for health. The increase in plastic waste inevitably determines its dispersion into the environment, where it undergoes various degradation processes, which lead to continuous fragmentation. UVB irradiation is one of the first causes of the ageing of these materials, which inevitably alters their structure. Particles that are increasingly smaller and have an increasingly negative surface charge can trigger biochemical and structural alterations within the cellular compartment, laying the foundations for establishing even important pathological events.

In the general population, exposure to MPs can occur directly (contact, inhalation, or ingestion) or indirectly (ascending of MPs in the trophic chain), and these particles can be excreted or accumulated in various tissues, increasing the toxicological risk dictated by exposure to different xenobiotics environmental. This phenomenon may cause different diseases, considering current estimates suggest that an individual ingests between 0.1 and 5 g of plastic material per week.

In conclusion, further studies would be necessary to evaluate the effects of MPs in vivo and also through biomonitoring studies to be performed on the general population in order to identify the long-term effects of this recent class of persistent pollutants.
